# Gaining insight into how women conceptualize satisfaction: Western Australian women’s perception of their maternity care experiences

**DOI:** 10.1186/s12884-015-0759-x

**Published:** 2016-02-04

**Authors:** Lucy Lewis, Yvonne L. Hauck, Fiona Ronchi, Caroline Crichton, Liana Waller

**Affiliations:** School of Nursing, Midwifery and Paramedicine, Curtin University, Bentley, Perth, 6102 Western Australia Australia; Department of Nursing and Midwifery Education and Research, King Edward Memorial Hospital, Subiaco, Perth, 6008 Western Australia Australia

**Keywords:** Maternal satisfaction, Mixed methods, Woman focused care

## Abstract

**Background:**

The concept of maternal satisfaction is challenging, as women’s and clinicians’ expectations and experiences can differ. Our aim was to investigate women’s experiences of maternity care in an urban tertiary obstetric setting, to gain insight into conceptualization of satisfaction across the childbirth continuum.

**Methods:**

This mixed method study was conducted at a public maternity hospital in Western Australia. A questionnaire was sent to 733 women two weeks post birth, which included an invitation for an audio-recorded, telephone interview. Frequency distributions and univariate comparisons were employed for quantitative data. Thematic analysis of interview transcripts was undertaken to extract common themes.

**Results:**

A total of 54 % (399 of 733) returned the questionnaire. Quantitative results indicated that women were less likely to feel: involved if they did not have a spontaneous vaginal birth (*P =* 0.020); supported by a midwife if they had a caesarean (*P* = <0.001); or supported by an obstetrician if they had a spontaneous vaginal birth (*P* = <0.001).

Qualitative findings emerged from 63 interviews which highlighted the influence that organization of care, resources and facilities had on women’s satisfaction. These paradigms unfolded as three broad themes constructed by four sub-themes, each illustrating a dichotomy of experiences. The first theme ‘how care was provided’ encompassed: familiar faces versus a different one every time and the best place to be as opposed to so disappointed. The second theme ‘attributes of staff’ included: above and beyond versus caring without caring and in good hands as opposed to handled incorrectly. The third theme ‘engaged in care’ incorporated: explained everything versus did not know why and had a choice as opposed to did not listen to my needs.

**Conclusions:**

Quantitative analysis confirmed that the majority of women surveyed were satisfied. Mode of birth influenced women’s perception of being involved with their birth. Being able to explore the diversity of women’s experiences in relation to satisfaction with their maternity care in an urban, tertiary obstetric setting has offered greater insight into what women value: a sensitive, respectful, shared relationship with competent clinicians who recognise and strive to provide woman focused care across the childbirth continuum.

## Background

Women’s satisfaction with maternity care is important to healthcare professionals, hospital administrators and policy makers [1, 2] as the feedback gleaned is used to improve maternity services [3] and inform decisions around the use of hospital resources [2, 4]. Besides the outcomes of maternal and infant morbidity and mortality, addressing components that constitute women’s satisfaction with maternity care should be a focus of maternity services in the 21^st^ century.

It is unusual for a woman to feel completely satisfied with every aspect of her care. More likely she will rank the quality of her care as satisfactory, but when asked to reflect on her experience she can often share what she liked and disliked [5]. Two decades ago a large Australian survey found that women experience greater satisfaction with their antenatal opposed to intrapartum care [6]. Other Australian research has found women who birth in the public sector were more likely to be satisfied than those birthing in the private sector, especially if they received professional support within 10 days post discharge [7]. Women who have increased obstetric intervention such as induction of labour are generally less satisfied with their care [8]. Indeed, a study comparing satisfaction with mode of birth found most women prefer a vaginal birth and that maternal satisfaction with vaginal birth was high [9].

A systematic review suggested continuous support from caregivers markedly improves maternal satisfaction [10]. This finding is unsurprising as continuous support has the capacity to improve comfort, emotional support, information and advocacy, thereby enhancing the perception of control [11]. Indeed when women evaluate their experiences five factors predominate: experiences that met or exceeded expectations [10, 12, 13]; staff qualities including quality of care and support [2, 3, 10, 14]; involvement with decision making [3, 6, 10, 15]; woman focused care [2]; and systems and faculties [2, 3, 16].

It is difficult to gauge how the issues raised by women impact their overall satisfaction [2], since research often reports responses to measures separately [3, 5, 14] rather than investigating components of satisfaction simultaneously or in clusters [3]. Traditionally, the focus of obstetric maternity care has been on outcomes such as morbidity and mortality, concepts which relate to physical not psychological safety [17]. Research has investigated satisfaction across the pregnancy and childbirth continuum, particularly during birth [1, 3, 13, 18, 19] using a variety of methods. Quantitative methodologies have dominated [19], where prescribed lists of categories are presented in measures, which may not be able to unravel the importance of an issue in relation to other aspects of care [2]. These quantitative studies struggle to illustrate the richness of women’s realities that could be revealed through qualitative designs. However, the latter provide limited guidance for policy makers as the perceptions of individual women are unique and generated themes can only be utilised to enhance knowledge of the phenomenon [13], but not as evidence to direct the focus of resources.

Although there is evidence around maternal satisfaction, in Australia there are gaps in our knowledge especially around identification of components that constitute women’s satisfaction within an urban, tertiary obstetric setting. In the absence of research, the aim of this study was to investigate women’s experiences of their maternity care within a tertiary obstetric hospital to gain insight into how women conceptualised satisfaction across the continuum of pregnancy, birth and one week post birth.

## Methods

### Design, participants and setting

A mixed methods design was used as it has been suggested that satisfaction surveys should preferably utilize a range of tools to avoid promoting the status quo [20]. Compared to single methods, mixed methods are ideally suited to provide insight and understanding into complex issues where further in-depth knowledge is required [21, 22]. Mixed methods have been described as an evolving research paradigm which build on triangulation between methods rather than within methods [23]. This methodology gives qualitative researchers the opportunity to utilize quantitative research to give a more comprehensive overview and deeper understanding of the investigated phenomenon [23]. By utilizing this methodology we were able to provide a more informative, complete and balanced overview of the research results.

This study was performed at the sole public tertiary obstetric hospital in Western Australia (WA) for women with complex pregnancies; recent data from the ‘Safe Tracking Obstetric Record Keeping’ database at the study centre found in 2012, 23 % of all births were preterm (less than 37 weeks gestation). In 2012 preterm birth occurred in 9 % of all births in WA [24]. The annual births at this public tertiary obstetric hospital are approximately 6,000. Between October and December 2013, English speaking women, who received scheduled antenatal care, birthed a live baby and were cared for by the visiting midwives service (VMS) on discharge from hospital were invited to participate.

At the study setting, women were booked for antenatal care by a midwife. Pregnancy care was subsequently provided by midwives and obstetricians, with women deemed to be high risk referred to a specialist obstetric clinic. Intrapartum care was centralised in labour and birth suite under a multidisciplinary team of midwives, obstetricians, paediatricians and anaesthetists. Women were discharged from the hospital to VMS, 24 hours after a vaginal birth or uncomplicated assisted birth and 72 hours after a caesarean birth.

### Recruitment and data collection

A total of 733 women were invited to participate through an information letter, accompanied by an in-house designed, two-page, 26 question, reply paid questionnaire, posted to their home two weeks post birth. Although the questionnaire was not validated, an earlier version had been used previously, administered at the same time post birth, for women having a scheduled caesarean [25]. Women were asked to provide information about themselves and their experiences of pregnancy, birth and the first week of their baby’s life. Returning a completed questionnaire was deemed implied consent. Questions covered who women received care and support from and the importance of continuity of care; utilising Likert scales and open ended questions.

An item was included inviting women to participate in a semi-structured, audio recorded, telephone interview with a research midwife, not involved with their clinical care, to share details about the care received. This option was removed after six weeks of data collection, when 18 % of women (63 of 342) had been interviewed and data saturation achieved with no new information being gleaned [26]. Three questions were asked during the interview: what did you like about your care? What didn’t you like about your care? and How could we improve the care offered? Telephone interviews lasted between five and 25 minutes. Verbatim transcripts of interviews and field notes were stored on a password protected computer in accordance with the National Health and Medical Research Council (NHMRC) guidelines [27]. Some women participating in this study were less than 18 years old and could be considered as young people who need consent from a parent or guardian [27]. However, as discussed in the NHMRC guidelines [27] these participants were considered as mature young women, as they were accessing their own healthcare and caring for their own child. Ethical approval was obtained from the Women and Newborn Health Service Human Research Ethics Committee (5740/EW) which adheres to the NHMRC guidelines.

### Data analysis

Quantitative data was analysed using frequency distributions to summarise categorical data (e.g. satisfaction with birth). Univariate comparisons between mode of birth (vaginal assisted and caesarean birth) were performed using *X*^2^ tests. *P*-values <0.05 were considered statistically significant. SPSS statistical software (version 21) was used.

Transcribed interviews were subjected to thematic analysis. Analysis was a continual process requiring the research team to become immersed in the data to deconstruct and extract common themes, patterns and similarities around the women’s experiences of their maternity care [28]. Explicit themes evolved from direct words or sentences [29]. Four members of the research team analysed a cross-section of transcripts and field notes ensuring each data source was reviewed by at least two members. The team met weekly over two months to negotiate, clarify and refine the findings. Any disagreements on interpretation were negotiated by referring back to the data. All the researchers were female, clinical or academic midwives, and with varying experiences of providing and receiving maternity care.

## Results

### Quantitative findings

Table 1 presents data for the 54 % (399 of 733) of women who returned a questionnaire. Although parity of the sample was similar, differences between parity and mode of birth were significant (*P* < 0.001). A higher proportion of women having a spontaneous vaginal birth were more likely to feel involved with their birth than the women having an assisted or caesarean birth (91 % vs 83 % and 81 %; *P =* 0.020). During their birth a higher proportion of women having a spontaneous vaginal or assisted birth felt supported by a midwife compared to women who had a caesarean birth (83 % versus 63 %; *P* = <0.001). During their birth a higher proportion of women having an assisted or caesarean birth compared to women having a spontaneous vaginal birth felt supported by an obstetrician (41 % and 36 % versus 9 %; *P* = <0.001). In the first week of the baby’s life a higher proportion of women having an assisted or caesarean birth compared to women having a spontaneous vaginal birth felt they received care from an obstetrician (32 % and 36 % versus 17 %; *P* = <0.001). The majority of women (96 %) would recommend the hospital to their family and friends.

**Table 1:** Demographic variables and satisfaction with care during pregnancy, birth and the first week of the baby’s life

Outcome	Spontaneous birth	Assisted birth	Caesarean birth	*P* Value	Total
	*n* = 195	*n* = 59	*n* = 145		*n* = 399
	*n* (%)	*n* (%)	*n* (%)		*n* (%)
Age (years)					
≤ 24 years	27 (14)	2 (3)	6 (4)	0.014	35 (9)
25–34	120 (61)	42 (71)	99 (68)		261 (65)
≥ 35	48 (25)	15 (25)	40 (28)		103 (26)
Parity					
Primiparous	72 (37)	45 (76)	69 (48)	<0.001	186 (47)
Multiparous	123 (63)	14 (24)	76 (52)		213 (53)
During pregnancy					
Important to have care same midwife	98 (53)	28 (48)	77 (55)	0.715	203 (53)
Received care from same midwife	50 (27)	14 (25)	43 (31)	0.609	107 (28)
Important to have care same obstetrician	55 (35)	15 (29)	37 (27)	0.338	107 (31)
Received care from same obstetrician	102 (61)	33 (63)	99 (72)	0.159	234 (66)
Agreed care in pregnancy met needs	175 (92)	55 (93)	126 (88)	0.324	356 (90)
The birth experience					
Felt involved with birth	173 (91)	49 (83)	114 (81)	0.020	336 (87)
Felt received midwifery support	160 (83)	48 (83)	91 (63)	<0.001	299 (76)
Felt received obstetric support	17 (9)	24 (41)	52 (36)	<0.001	93 (23)
First week of baby’s life					
Felt received care from midwife	156 (80)	53 (90)	125 (87)	0.121	334 (84)
Felt received care from obstetrician	33 (17)	19 (32)	52 (36)	<0.001	104 (26)
Recommend King Edward Memorial Hospital to family and friends	189 (97)	54 (92)	141 (97)	0.833	384 (96)

Variables did not always add up to *n* = 399 due to missing values for some responses

Of the *n* = 145 women having a caesarean birth *n* = 80 (55 %) had an elective caesarean and *n* = 65 (45 %) had an emergency caesarean

### Qualitative findings

Of the 63 women interviewed 51 % (32) were multiparous. Over half (56 % or 35) were 25 to 34 years old. A total of 48 % (30) had a spontaneous vaginal birth, with 8 % (5) having an assisted birth and 44 % (28) having a caesarean.

Exploration of women’s experiences of their maternity care provided insight into how women conceptualised satisfaction. Three main themes emerged from the data: how care is provided; attributes of staff; and engaged in care (Table 2). Findings are supported with direct quotes from women’s stories. A coding system (P1 to P342) was used for each woman to ensure their confidentiality and privacy would be respected. Additionally, each quote was allocated a postfix to indicate parity (‘P’ for primiparous and ‘M’ for multiparous) and mode of delivery (‘V’ for a vaginal birth and ‘C’ for a caesarean birth).

**Table 2:** Women’s conceptualization of satisfaction with their maternity care

How care is provided	
Familiar faces	A different one every time.
The best place to be	So disappointed
Attributes of staff	
Above and beyond	Caring without caring
In good hands	Handled incorrectly
Engaged in care	
Explained everything	Did not know why
Had a choice	Did not listen to my needs

### How care is provided

Our analysis revealed the influence that organisation of care, resources and facilities had on women’s satisfaction. How care was provided was reflected by four sub-themes that illustrated a dichotomy of experiences. Familiar faces versus a different one every time and the best place to be as opposed to so disappointed (Table 2).

#### Familiar faces

Women acknowledged knowing their caregivers enhanced their experience. Some women knew they *‘felt quite comfortable the whole time because there was a familiar face there’* (P273^P,C^). One woman had the insight to recognise *‘I really liked that it was the same midwife, being able to build rapport*’ (P272^M,V^). Another appreciated her midwifery care was organised to promote continuity of carer. ‘*I’ve seen the same midwife come back day after day…they were trying really hard to have the same midwives come in after I’d had him [baby]*’ (P29^M,C^). Continuity of carer did not have to occur across the continuum of pregnancy and birth. For example one woman noted how the stress of her caesarean birth was negated by having the same midwife stay with her for this event: ‘*It was good to have continuation of support while we were waiting to go into theatre all the way through to we were out*’ (P178^P,C^).

#### A different one every time

Not having any degree of continuity of carer was problematic. Different carers were challenging to women who had ‘*to repeat*’ (P4^M,V^, P75^M,V^, P97^P,V^) their histories. Being exposed to different carers led one woman to reflect ‘*there were holes in some things, but I suppose that’s because there were so many different people involved in my care*’ (P38^P,V^). Scenarios illustrated frustration with the perceived inability to organise continuity of carer. One woman voiced ‘*it wasn’t a team approach to planning the birth because I saw a different midwife each time [in pregnancy]…I would have liked continuity of carer*’ (P303^P,V^). Another reflected ‘*I would have liked the VBAC [Vaginal Birth After Caesarean clinic] midwives to be involved in the labour. They were so invested and involved in my antenatal care…it would have been nice if they could have been there to help support me when the birth happened*’ (P96^M,C^).

#### The best place to be

When facilities were mentioned it was the access to specific professions and all the ‘*extra people*’ (P29^M,C^), ‘*physio and breast feeding clinics*’ (P272^M,V^) rather than the physical environment that was central, leading one woman to describe the hospital as ‘*the best place to be to have a baby*’ (P87^M,C^). Another recounted she had ‘*a few people visiting my room, different professions, physiotherapy and the things I could do for my body after birth… I thought it’s a really good option for mums*’ (P330^P,C^).

#### So disappointed

Narratives illustrating disappointment were triggered by the amount of one on one time midwives could spend with women following their birth. One woman was ‘*so disappointed with the care on the ward I begged to be discharged as I firmly believe care at home would be better*’ (P312^M,V^). Another recounted ‘*on one occasion and some others the midwife left to get something and we were forgotten about*’ (P2^P,C^). Disappointment was compounded when women felt *‘chucked out of the door’* (P61^M,V^) and ‘*I was really rushed off the ward to go home…I had gestational diabetes and the baby’s sugar levels hadn’t quite stabilised*’ (P261^M,V^). Others were disappointed ‘*they [VMS] visited me once and I was expecting more’* (P151^P,C^). Another recounting she had ‘*a vaginal birth and I know the general policy is 24 hour [discharge] but as a first time mum we sort of got home and didn’t know*’ *[how to care for the baby]* (P18^P,V^).

### Attributes of staff

Personal characteristics of individual staff influenced women’s satisfaction. The second theme ‘attributes of staff’ was dependent on four sub-themes: above and beyond versus caring without caring; and in good hands as opposed to handled incorrectly (Table 2).

#### Above and beyond

Many personal attributes of staff were described as positive. For example, women were ‘*blown away*’ (P219^P,V^, P154^M,C^) by midwifery staff described as going ‘*above and beyond*’ (P38^P,V^, P96^M,C^) and ‘*fantastic*’ (P18^P,V^, P261^M,V^, P276^M,V^). One woman noted ‘*even though they were overloaded they still made time to make you feel like you weren’t just a number*’ (P313^M,C^). Personalised care was important as it was viewed as more than just completion of a clinical task, additionally, having a positive attitude was valued. One woman’s scenario described how a positive midwife made all the difference with her inability to breastfeed:

‘*One of the midwives gave me a massive hug. She said look you’re doing a good job. So come on cheer yourself up, your daughter’s fine….it was the middle of the night and my husband wasn’t there and I just really needed that at that time because previously I’d really felt like a failure*’ (P178^P,C^).

#### Caring without caring

A different experience was noted for those women who revealed they had higher expectations of the midwives they encountered. One woman described her midwife as ‘*unhelpful to the point of hostile*’ (P69^P,V^). Certain skills were expected, one woman felt even a basic level of care was not possible as her midwife ‘*didn’t make conversation and didn’t seem to have that instinct of natural care you have to have as a midwife*’ (P209^P,V^). Not only were some midwives perceived as unhelpful and uncaring but their influence was demoralising:

‘*There were a couple of midwives on the ward after I’d had her [my baby] that were the worst people I had ever met and really made me feel like the most stupid person in the world. Some of the midwives were in the wrong job, you shouldn’t be a midwife if you’re caring without caring*’ (P178^P,C^).

#### In good hands

Being ‘*in good hands*’ (P62^M,C^, P140^P,C^) was interlinked with helping women feel ‘*safe*’ (P61^M,V^, P69^M,V^, P219^P,V^) especially when they were vulnerable. ‘*Post unplanned caesarean I was weak and vulnerable. The care helped me feel secure, heal and it was crucially helpful’* (P15^P,C^). Being ‘*secure and confident with the standard of care*’ (P289) was paramount. One woman reflected ‘*they were really right on top of everything I needed to know*’ (P276^M,V^). Another recounted ‘*she was another one of those you know really top notch midwives who obviously knows her job*’ (P38^P,V^). One woman had the insight to recognise ‘*they knew exactly what they were doing I just trusted them and just felt at ease*’ (P125^P,V^).

#### Handled incorrectly

At the other end of the spectrum, women shared examples of unmet expectations and perceived they were ‘*handled inappropriately*’ (P69^P,V^) which created negative emotions which diminished their experience. The uncertainty of what was happening surrounding caesarean birth especially caused distress. One woman recounted her ‘*surgeon was shouting and panicky-that worried m*e’ (P63^P,C^). Being cared for by staff who did not describe what was happening caused distress. ‘*One minute I was told we were waiting for a surgeon and a minute later I was told they could see the bum [baby’s buttock] but at no point was I told they had begun [my caesarean]*’ (P57^P,C^). Being ignored was especially distressing, as one woman narrated ‘*I went into my own zone to cope…I could feel what they were doing….I remember I felt like I was going to die. The theatre nurse saying “If you behave like that I cannot help you"* (P253^P,C^).

### Engaged in care

The third theme around the conceptualisation of satisfaction revealed the influence that listening, explanation and choice had on women’s experiences. Again, being engaged in care was dependant on four dichotomous sub-themes: explained everything versus did not know why; and had a choice as opposed to did not listen to my needs (Table 2).

#### Explained everything

Women valued ‘*being kept informed*’ (P127^P,V^, P219^P,V^, P310^P,V^). To illustrate, one woman appreciated she was told ‘*the truth without sugar coating*’ (P83^M,C^), whilst another reflected she liked not being ‘*fobbed off or patronised*’ (P119^M,V^). Having staff who ‘*really explained everything’* (P315^M,V^) was reassuring for one multiparous woman. Another woman narrated ‘*they communicated very clearly with me what was going on so I didn’t feel like I was left in the dark, I was well informed*’ (P268^M,V^). Having ‘*any questions that I had answered*’ (P196) was important. One woman’s scenario described how when she had ‘*questions that they couldn’t answer they always appropriately and promptly consulted other people they thought would have answers for me, I thought that was fine*’ (P69^P,V^).

#### Did not know why

Women shared how they needed clarification regarding care around their birth and baby’s wellbeing. Not knowing caused disappointment. ‘*I had wanted a natural [birth] but was told I needed a caesarean with not great explanation as to why other options were not being tried*’ (P78^P,C^) and ‘*I had put on my birth plan for him not to be wrapped up and be given skin to skin. That didn’t quite go to plan… I don’t know what the reason was*’ (P118^P,C^). It was apparent that not knowing caused anxiety especially for labouring women. ‘*I needed an emergency caesarean they did not inform or ask me, no idea why….no one was speaking to me*’ (P253^P,C^) and ‘*the doctors came for forceps and I did not know why*’ (P248^P,V^).

#### Had a choice

Being given choice assisted women to decide what was best for them. ‘*I was told I could ... use the fitness ball, bean bag. I could be on the floor, standing up, in the shower…I could just try different things and see what felt best for me*’ (P272^M,V^). Women’s narratives illustrated those who ‘*had a choice*’ (P303^M,C^) felt ‘*in control a whole lot more*’ (P313^M,C^). Stories revealing choice resulted in respect which enhanced perceptions of control. Not having an imposed agenda fostered mutual respect. ‘*I was given the opportunity to try that’s probably the main thing’* (P196^M,C^) and ‘*I never felt at any stage my decisions weren’t respect*ed’ (P69^P,V^).

#### Did not listen to my needs

Contrary to having choice, not being able to negotiate care triggered confusion. ‘*They didn’t listen to my needs. It was all really confusing*’ (P8^P,C^). The margin between not being listened to and being ignored was blurred, preventing care from being shared between caregivers and women. One mother recounting that her ‘*birth plan was completely ignored*’ (P5^P,C^) and another voicing ‘*they didn’t let me have bub [baby] with me in recovery…I was very disappointe*d’ (P29^M,C^). Women’s narratives revealed a relationship with caregivers reflecting not being listened to and a perception of being coerced. One woman recounted her midwife stating ‘*this is what we do here then I’d say well I don’t really want to do that*’ (P315^M,V^) whilst another voiced ‘*there were a few pushy midwives trying to get me to do it in a way I didn’t really like*’ (P334^P,V^).

## Discussion

This mixed methods analysis facilitated the exploration of women’s experiences of maternity care, allowing insight into how they conceptualized satisfaction within an urban, tertiary obstetric setting. Quantitative analysis found the majority of women would recommend the hospital to their family and friends. Those having a spontaneous vaginal birth were more likely to feel involved with their birth than those having an assisted or caesarean birth. However, the majority of women felt involved with their birth. Although the qualitative sub-themes were presented separately they were interrelated, revealing positive and negative paradigms. Our discussion will focus around; how our research resonates with the work of others, how mixed methods enhanced our research and discussion around selected opposing sub-themes.

Much of what the study found echoes the work of others internationally around the physical environment [3], interpersonal care [3, 16, 19], information giving [3, 16, 19] and the role of decision making increasing women’s perception of control [3, 16, 19]. Our study also found similarities with other Australian research, specifically in relation to how women conceptualize continuity [2, 15], support during birth [2, 5, 15, 30] and issues around meeting childbirth expectations [2, 13, 30]. However, this is the first Australian study performed solely within a tertiary obstetric setting, which incorporates mixed methods across the childbirth continuum and one week post birth. Providing a connection between the quantitative and qualitative research was challenging, but enabled the researchers to answer questions that could not be answered by single methods alone [20], such as the richness of birth experiences.

Although the qualitative results dominate this manuscript, we were able to use quantitative results from the questionnaire to highlight satisfaction with care during pregnancy, birth and the first week of the baby’s life for subgroups of women based on mode of birth. We acknowledge the quantitative methods provided limited opportunity to contextualize the women’s experiences, but they did enable our research team to utilize both numbers and words to conceptualize maternal satisfaction [21, 23]. Quantitative analysis of mode of birth, also enabled us to reinforce our cohort was representative of women giving birth at the study centre in 2011, where 35 % delivered by caesarean and 65 % had a vaginal birth [24].

The synopsis of women dissatisfied with their care, captured dimensions where difficulties were encountered, especially the impression of not having their wishes listened to so their expectations of care could not be addressed. This was illustrated through the themes of ‘caring without caring’ and being ‘handled incorrectly’. Others have described the negative impact when communication issues result in expectations not be respected [1, 5, 31, 32]. Research suggests women may actively construct schema of their maternity care expectations, which are used retrospectively to evaluate their experiences [30]. It has also been found that although women may have multiple expectations for birth, specific expectations are prioritized.

It has been suggested that withholding information can disempower women [19], so if things do not unfold smoothly they may perceive a profound loss of control. In our study this was illustrated through the recollections of women who ‘did not know why’, especially those who were trying to make sense of a caesarean birth or intervention where they perceived clinicians had not listened to their needs. Others have suggested that a woman’s personality can hinder or assist adaptation to life events [32–34]. Indeed, women who are confident to question their clinicians about the why and how, may be more likely to prompt shared decision making with their clinicians [34]. This was demonstrated by the sub-theme ‘explained’ everything, where having ones questions answered, enabled women to make sense of interventions. This ability to question enhanced their ability to navigate themselves through complex maternity systems [35], so women were less likely to perceive they ‘did not know why’, or feel caregivers ‘did not listen to their needs’.

Overall the sub-themes of the WA women who were satisfied illustrated they had everything explained, by caregivers who went above and beyond. The tertiary obstetric hospital environment did not diminish their experience, as they perceived wide spectrums of support from their caregivers [10, 36], highlighted by the sub-theme ‘the best place to be’. These reflections show the stereotyped difficulty [16, 19] around the provision of care for complex pregnancies can be overridden. Women’s expectations of maternity care were not only met but exceeded as their individual, idiosyncratic, contextual factors were acknowledged enhancing their perception of control [12, 37, 38]. Others have reported similar narratives [2, 19, 39] suggesting these common threads reflect the constituents of women centred care [2].

Conversely when the tertiary obstetric hospital environment hospital environment did diminish women’s satisfaction with maternity care, it was shaped by sub-themes such ‘did not listen to my needs’ and ‘a different one every time’. Indeed, a recent randomised controlled study compared group based antenatal care to standard care [40] finding group based care fostered better engagement with midwives and less deficiency with information giving around care. The decreased involvement of women in their care decisions [41] has been shown to negatively affect their satisfaction [23, 42]. Being coerced to accept clinical advice caused tension, especially if the advice negated woman’s requests entirely, was presented without rationale and did not reflect the woman’s birth plan. Women’s stories revealed the power of maternity clinicians to shape the ethical and moral environment of provision of care [43]. Encountering these negative experiences jeopardised women’s satisfaction with their maternity care.

### Limitations

There were a number of limitations. Women in this study received care within one tertiary maternity hospital, which provides care for a significant proportion of high risk pregnancies within one Australian state. The optimum time for recall around birth experience is dependent on personal preference, we acknowledge this could have had an impact in relation to the response rate and findings. The self-selection of women for qualitative interview could have resulted in responders at both extremes of satisfaction. The sample comprised English speaking women giving birth to a live infant and is not representative of all birthing women in WA. Although all women surveyed had birthed a live infant, we were unaware if they had birthed a healthy child, willingness to disclose this information during interview was the woman’s choice. Therefore the context of the study must be considered when interpreting generalizability of the findings to other settings.

## Conclusion

The concept of maternal satisfaction is challenging, as women’s and clinician’s expectations and experiences can differ. The quantitative analysis in this mixed method design confirmed that the majority of women surveyed were satisfied. Mode of birth influenced women’s’ perception of being involved with their birth. However, qualitative data provided rich insight into the complexities around how women conceptualise satisfaction. Being able to explore the diversity of women’s experiences in relation to satisfaction with their maternity care in an urban, tertiary obstetric setting has offered greater insight into what women value; a sensitive, respectful, shared relationship with competent clinicians who recognise and strive to provide woman focused care across the childbirth continuum.
